# Prevalence of *Helicobacter pylori* among Sudanese patients diagnosed with colon polyps and colon cancer using immunohistochemistry technique

**DOI:** 10.1186/s13104-020-05159-2

**Published:** 2020-07-06

**Authors:** Aziza K. Mohamed, Nazik M. Elhassan, Zahra A. Awhag, Fadwa S. Ali, Eman T. Ali, Najwa A. Mhmoud, Emmanuel E. Siddig, Rowa Hassan, Eiman S. Ahmed, Azam Fattahi, Ayman Ahmed, Mohamed S. Muneer, Hussam A. Osman, Nouh S. Mohamed, Ali M. M. Edris

**Affiliations:** 1 Department of Histopathology and Cytology, Faculty of Medical Laboratory Sciences, University of Khartoum, Khartoum, Sudan; 2 Department of Histopathology and Cytology, Faculty of Medical Laboratory Sciences, National University, Sudan, Khartoum, Sudan; 3 Mycetoma Research Center, University of Khartoum, Khartoum, Sudan; 4 Department of Microbiology and Immunology, Faculty of Medical Laboratory Sciences, University of Khartoum, Khartoum, Sudan; 5 School of Medicine, Nile University, Khartoum, Sudan; 6 Department of Histopathology and Cytology, Alfarrabi College for Science and Technology, Khartoum, Sudan; 7 Center for Research and Training in Skin Disease and Leprosy, Tehran University of Medical Sciences, Tehran, Iran; 8 Department of Parasitology and Medical Entomology, Institute of Endemic Diseases, University of Khartoum, Khartoum, Sudan; 9 Department of Neurology, Mayo Clinic, Jacksonville, FL USA; 10 Department of Radiology, Mayo Clinic, Jacksonville, FL USA; 11 Department of Internal Medicine, Faculty of Medicine, University of Khartoum, Khartoum, Sudan; 12 Biomedical Research Laboratory, Ahfad University for Women, Omdurman, Sudan; 13 Molecular Biology Department, Alfarrabi College for sciences and Technology, Khartoum, Sudan; 14 Department of Parasitology and Medical Entomology, Faculty of Medicine, Sinnar University, Sinnar, Sudan; 15 Department of Parasitology and Medical Entomology, Faculty of Medical Laboratory Sciences, Nile University, Khartoum, Sudan; 16 Faculty of Applied Medical Sciences, University of Bisha, Bisha, Kingdom of Saudi Arabia

**Keywords:** Colon polyps, Colon cancer, *Helicobacter pylori*, Immunohistochemistry

## Abstract

**Objectives:**

Infection with the bacteria *Helicobacter pylori* has been classified as class one carcinogen associated with increasing susceptibility of gastritis and gastric carcinoma. This study is aiming at investigating the prevalence of *H. pylori* among colon polyps and colon cancer patients. A descriptive cross-sectional hospital-based study was conducted between February and June 2017. Sixty-nine formalin-fixed paraffin blocks collected from colon polyps and colon cancer patients to detect *H. pylori* using immunohistochemistry technique.

**Results:**

Of the 69 patients included in the study, 39 (56.5%) males and 30 (43.5%) were females, their age ranged from 21 to 80 years with a mean age of 47.1 ± 19.7. Of the 69 colon polyps and colon cancer patients, 44 (63.8%) were diagnosed as adenocarcinoma, 10 (14.5%) colitis, 15 (21.7%) juvenile polyposis syndrome. The results of immunohistochemistry technique showed the presence of 16 (23.2%) positive patients for *H. pylori* infection. Of these 16, 13 (81.3%) patients were diagnosed with adenocarcinoma and 3 (18.7%) patients were diagnosed with juvenile polyps. The results of *H. pylori* detection among the different colon polyps and colon cancer patients were showing a statistically significant association for *H. pylori* infection and adenocarcinoma, P value 0.028.

## Introduction

Colorectal cancer (CRC) is accounted as the third most common malignancy and the third most common cause of death due to cancer in both men and women in the US [1]. CRC mostly arises from adenomatous polyps (adenomas) and from hyperplastic polyps [2, 3]. Early diagnosis and surgical removal of these polyps have associated with the decreased in the incidence of mortality [4–6]. Therefore, clinicians started to focus in recent years on the prevention measures that decrease the incidence of developing CRC; although, researchers started to explore the role of several infectious organism and their ability to increase or induce CRC [7–10]. For instance, many epidemiological studies have linked the infection of *Helicobacter pylori* to colorectal neoplasm either through high prevalence of *H. pylori* seropositivity among CRC or colorectal polyp patients [11–13], or through the presence of bacterial products and their trophic effects on colon mucosa [14–17]. Moreover, few studies have linked the presence of *H. pylori* in the stomach or colon with colon cancer and/or polyps [18–23].

It is well known that *H. pylori* predisposes to develop gastric cancer precursor lesions, thus it has been classified as class 1 carcinogen [24]. A recent meta-analysis correlating between *H. pylori* and extra-gastric malignancies revealed a statistically significant relationship of *H. pylori* infection with both colon cancer and polyps [25]. Also, *H. pylori* infection linked with colorectal lesions appeared to be more common in African Americans compared to the Caucasian population in the US [26, 27].

Epidemiological studies have confirmed a causal relationship between *H. pylori* and gastric cancer, and the colonic phenotype of *H. pylori*-related intestinal metaplasia (IM) [28]. Thus, association of *H. pylori* in various gastrointestinal cancers has been investigated, moreover, *Helicobacter* DNAs were prevalent in more than 50% of hepatobiliary cancer cases [29]. *Helicobacter* species, which may colonize the biliary tract, have been implicated as a possible cause of hepatobiliary diseases ranging from chronic cholecystitis and primary sclerosing cholangitis to gall-bladder carcinoma and primary hepatic carcinomas [30]. Therefore, the hypothesis that *H. pylori* would also be associated with colon lesions needs to be investigated. In Sudan, no reports addressing this manner were existed. Previous studies investigating the seroprevalence of *H. pylori* among Sudanese using ELISA and rapid immunochromatographic tests for the detection of *H. pylori* IgM and IgG antibodies, reported a seroprevalence ranging from 20% up to 70% [31–34]. Therefore, the aim of this study was to investigate the presence of *H. pylori* infections among Sudanese patients diagnosed with colon polyps and colon cancer and to correlate between its presence and the type of the lesions.

## Main text

### Materials and methods

#### Sample and data collection

This is a preliminary, descriptive study aimed to investigate the frequency of *H. pylori* infections among Sudanese patients diagnosed with colon cancer. Data were collected from 69 patients attended the National Laboratory and Alrahma Laboratory between February and June 2017.

During colonoscopy, presence of abnormal tissue, such as clumps of cells; polyps, formed on the inside of the colon, the pathologists obtained colon biopsies. Colon biopsies were fixed with formalin and processed into paraffin embedded blocks. Formalin-fixed paraffin blocks were used for the immunohistochemical detection of *H. pylori*. Ethical approval was previously obtained by the pathologists of each hospital before colon polyps’ biopsies were taken.

#### Preparation of the formalin fixed paraffin blocks

Four sections from each formalin-fixed paraffin block to increase detection sensitivity were obtained with a thickness of 4 µm using Rotary microtome (LEICA RM2125RT). All sections were de-waxed with two changes of Xylene for 3 min and then dehydrated in descending concentrations of Methanol starting from absolute Methanol through 90% and lastly, a concentration of 70% for 2 min in each concentration, and then washed using distilled water.

#### Immunohistochemistry diagnosis

Immunohistochemistry diagnosis was performed on all the obtained sections. Known gastric sections containing *H. pylori* infection was used as positive and negative controls; for the negative control the primary antibody incubation step was omitted. All sections were pretreated to retrieve antigens at 97 °C for 10 min in citrate buffer solution, and then sections were blocked by 3% Hydrogen peroxide and absolute Methanol for 20 min at humidified chamber. Afterward, sections were blocked into Bovine serum Albumin (Thermo Fisher Scientific, Germany). A rabbit polyclonal antibody ULC3R (BioGenex, USA) (prepared from tissue culture supernatant diluted in PBS, pH 7.6 containing 5% BSA and 0.09% Sodium Azaide) against *H. pylori* was applied for 40 min, then washed in buffer solution for 5 min. Then, a polymer solution was applied for 15 min, and washed in buffer solution for 5 min. Chromogen solution was added for 10 min, and washed in distilled water. Finally, Mayer’s Hematoxylin was added for 2 min, and then, sections were blued using running distilled water for 5 min. After bluing, sections were dehydrated, cleared, and mounted in DPX. Prepared sections were investigated microscopically by two experts’ pathologists blindly without knowing the duplication of slides sections of each patient using X40 lens. Results were recorded into categories of positive and negative results; the dot like shape denoted the coccoid form of the organism as describe previously [35–38].

#### Statistical analysis

Descriptive data were analyzed using the Statistical Package for Social Science (SPSS-v20). Pearson Chi-square test was used to test the association of *H. pylori* infection with the different types of lesions. A P value < 0.05 was considered as a statistically significant.

### Results

Of the 69 patients, there were 30 (43.5%) females and 39 (56.5%) males, their ages ranged from 21 to 80 years with a mean age 47.1 ± 19.8. 44 (63.8%) patients were diagnosed with adenocarcinoma, 10 (14.5%) patients were colitis, and 15 (21.7%) patients were juvenile polyposis syndrome. No statistically significant was observed for the association of gender and the pathological condition of each patient, P value = 0.649. Out of the 69 patients, 16 (23.2%) patients were positive for *H. pylori* infection. These 16 positive patients included; 13 (81.3%) patients diagnosed with adenocarcinoma and 3 (18.7%) patients diagnosed with juvenile polyps. The correlation between presence of *H. pylori* infection and the histopathological condition of patients were positively correlated (P value 0.028) (Table 1).

**Table 1:** Shows the correlation between gender, immunohistochemistry detection of *H. pylori* with the histopathological diagnosis

	Histopathological Diagnosis			Total	P value
	Adenocarcinoma	Juvenile polyposis syndrome	Colitis		
Gender					
Male	23 (58.9%)	10 (25.6%)	6 (15.5%)	39 (56.5%)	0.649
Female	21 (70.0%)	5 (16.7%)	4 (13.3%)	30 (43.5%)	
Total	44 (63.8%)	15 (21.7%)	10 (14.5%)	69 (100%)	
Immunohistochemistry of *H. pylori*					
Negative	31 (58.5%)	12 (22.6%)	10 (18.9%)	53 (76.8%)	0.028
Positive	13 (81.3%)	3 (18.7%)	0 (0.0%)	16 (23.2%)	
Total	44 (63.8%)	15 (21.7%)	10 (14.5%)	69 (100%)	

**Fig. 1:**
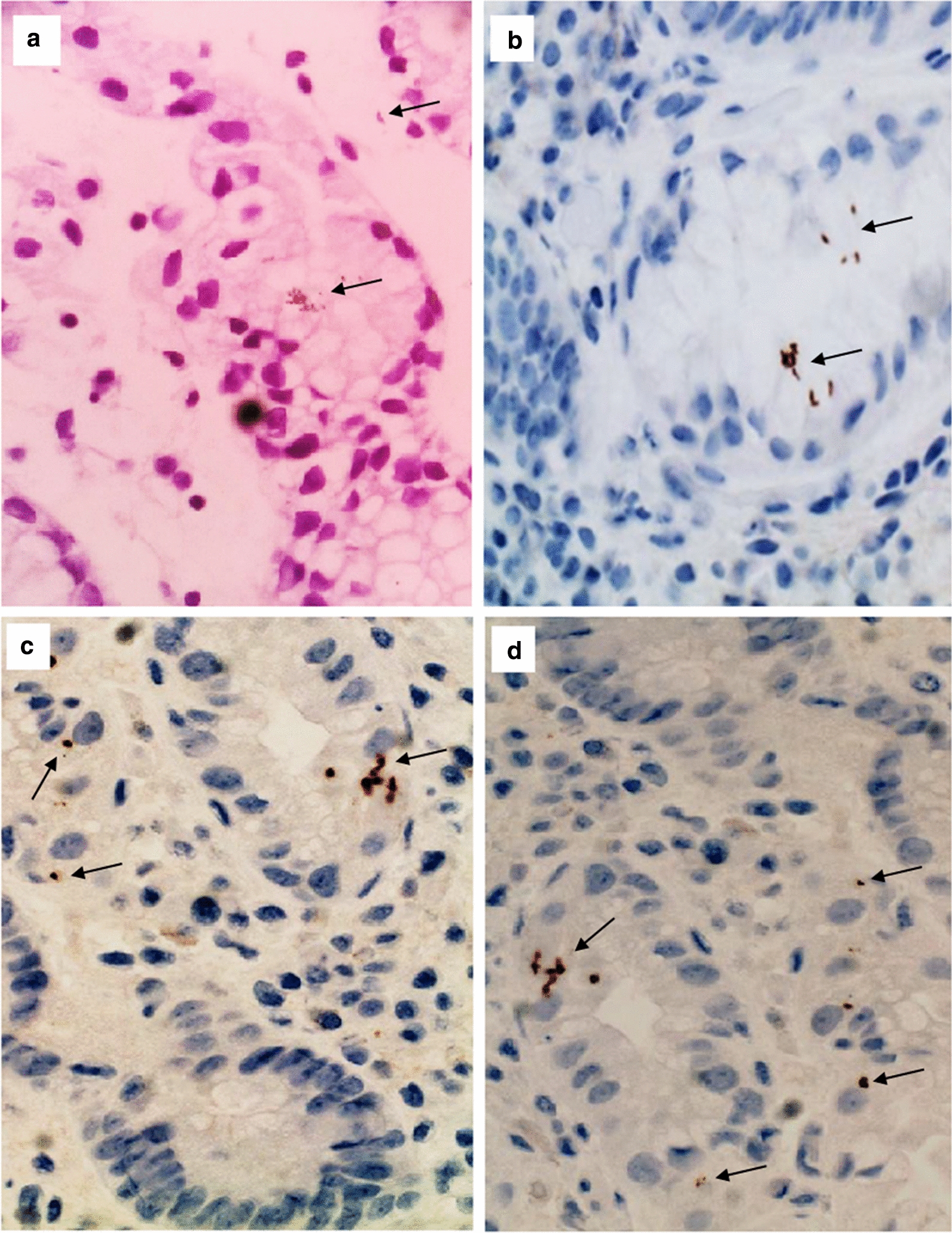
Detection of *H. pylori* using IHC staining technique. **a** Indicates positive control tissue section of known gastric biopsy infected with *H. pylori*. **b**–**d** Indicates tissue sections of patients diagnosed with colon polyps and colon cancer. Black arrows indicate *H. pylori* stained brown in color and take a dot and small curved shapes in different sizes

In respect to Immunohistochemistry diagnosis, the bacteria were prominent and easier to detect in the immune-stained sections in several patterns including organisms attached to the epithelial cells or within the superficial mucus. And in some cases, the bacteria were masked by inspissated mucus or being positioned flat and closely opposed to the epithelial surface. Regarding the morphological appearance of the organism; *H. pylori* stained brown in color and take a dot and small curved shape in different sizes (Fig. 1).

### Discussion

The exact role of *H. pylori* in the induction of colon cancer is still a debate between the scientific researcher communities; this is attributed to the controversial results obtained. In previous studies, *H. pylori* were linked to the development of gastric cancer [39], while others reported paradoxical results showing no association between *H. pylori* and gastric cancer susceptibility [22, 40, 41]. However, reports from Sudan regarding the possible link between *H. pylori* and colon cancer are scarce. Therefore, in the present study, we examined the presence of *H. pylori* using immunohistochemistry technique on colon polyps and colon cancer lesions of Sudanese patients underwent colonoscopy.

The results obtained from this study showed a positive correlation between the presence of *H. pylori* infection and the histopathological diagnosis, as *H. pylori* was prevalent in higher frequency in patients diagnosed with adenocarcinoma compared to those diagnosed as juvenile polyposis syndrome, and the result was statistically significant. This result also agrees with studies conducted by Jones et al. and Grahn et al. they investigated the presence of *H. pylori* among 59 patients diagnosed with colon adenocarcinomas using immunohistochemistry technique, and 77 colon and rectum cancer patients using molecular technique, correspondingly [20, 39]. Jones et al., reported that *H. pylori* were detected in 10/59 adenocarcinoma cases which represent about 16.9% of the total cancer cases studied [39]. While, Grahn et al. showed that *H. pylori* were present in 27% of the patients; among the studied colon cancer, *H. pylori* were present in 11/42 (26%) patients [20].

Although, several studies failed to demonstrate any association between *H. pylori* and colon cancer, or even if this microorganism can colonize the colon [42–46]. This could only be attributed to the ability of demonstrating the *H. pylori* bacteria, which was achieved by the aid of the immunohistochemistry technique that allowed a better localization of *H. pylori* within the various colon lesions of colitis, polyps, and adenocarcinoma included in this study.

Interestingly, several theories were proposed regarding the exact role by which *H. pylori* induced colon cancer, one hypothesis is that colon cancer can be induced by toxins produced by *H. pylori*; however, this theory was based only on serological data [22, 40, 41, 47]. Furthermore, some studies showed that colitis and colon cancer were also developed in experimental mice models infected with *H. hepaticus* [46]. Accordingly, the development of colon cancer seems most likely due to the interaction between toxins produced by the bacteria and the immune cells of the mice [46]. Therefore, the results we obtained from our study showing that *H. pylori* were present, nevertheless, it means that *H. pylori* infection is responsible for the induction and the development of colon cancer, since the presence of *H. pylori* could be encountered as post-cancer incidence. This however, still requires more complicated experimental studies to investigate this hypothesis; yet, the role of *H. pylori* cannot be excluded due to this hypothesis. Therefore, this preliminary report needs further advanced experimental investigations to enable the determination on the exact mechanisms by which *H. pylori* can induce colon cancer.

### Conclusion

This study was able to demonstrate the presence of *H. pylori* in colon polyps and colon cancer using immunohistochemistry marker, besides the significant association in the presence of *H. pylori* with colon adenocarcinoma, indeed further studies are required to elaborate more in-depth about the exact role of *H. pylori* in the development of colon cancer.

## Limitations

- In this study the sample size studied was relatively small, but still a significant association was observed. A bigger sample size of colon cancer lesions and benign colon lesions; non cancer lesions, should be included in future studies to determine the significant association of *H. pylori* with adenocarcinoma among the Sudanese patients diagnosed with colon cancer.

## Data Availability

The datasets used and/or analyzed during the current study are available from the corresponding author on reasonable request.

## Abbreviations

DNA: Deoxyribonucleic acid
CRC: Colorectal cancer
IM: Intestinal metaplasia
